# Specific Low/Endogenous Replication Stress Response Protects Genomic Stability via Controlled ROS Production in an Adaptive Way and Is Dysregulated in Transformed Cells

**DOI:** 10.3390/cells14151183

**Published:** 2025-07-31

**Authors:** Bernard S. Lopez

**Affiliations:** Université Paris-Cité, INSERM U1016, UMR 8104 CNRS, Institut Cochin, 75014 Paris, France; bernard.lopez@inserm.fr

**Keywords:** DNA damage response, ROS, replication stress, genetic instability, NF-κB, PARP1

## Abstract

Cells are assaulted daily by stresses that jeopardize genome integrity. Primary human cells adapt their response to the intensity of replication stress (RS) in a diphasic manner: below a stress threshold, the canonical DNA damage response (cDDR) is not activated, but a noncanonical cellular response, low-level stress-DDR (LoL-DDR), has recently been described. LoL-DDR prevents the accumulation of premutagenic oxidized bases (8-oxoguanine) through the production of ROS in an adaptive way. The production of RS-induced ROS (RIR) is tightly controlled: RIR are excluded from the nucleus and are produced by the NADPH oxidases *DUOX1/DUOX2*, which are controlled by NF-κB and PARP1; then, RIR activate the FOXO1-detoxifying pathway. Increasing the intensity of RS suppresses RIR via p53 and ATM. Notably, LoL-DDR is dysregulated in cancer cell lines, in which RIR are not produced by NADPH oxidases, are not detoxified under high-level stress, and favor the accumulation of 8-oxoguanine. LoL-DDR dysregulation occurred at an early stage of cancer progression in an in vitro model. Since, conversely, ROS trigger RS, this establishes a vicious cycle that continuously jeopardizes genome integrity, fueling tumorigenesis. These data reveal a novel type of ROS-controlled DNA damage response and demonstrate the fine-tuning of the cellular response to stress. The effects on genomic stability and carcinogenesis are discussed here.

## 1. Introduction

Genotoxic stress can lead to cell death, senescence, inflammation, genetic instability/mutagenesis, premature aging, and oncogenesis, and genetic instability is a hallmark of aging and cancer [1,2,3,4,5,6].

Cells are continuously confronted with stresses that compromise the integrity of their genome. The sources of these attacks on genomic stability can be exogenous, such as radiation (UV or ionizing) or chemical agents (pollution, tobacco, etc.), or endogenous, such as reactive oxygen species (ROS) or replication stress. Indeed, ROS can be spontaneous byproducts of cell metabolism and can generate premutagenic bases in DNA that can also alter replication dynamics [7,8,9]. Replication fork progression can be hindered by endogenous obstacles such as conflicts with transcription, proteins tightly bound to DNA, chemical adducts on the DNA, endogenous damage, and DNA structures that are difficult to replicate [10,11,12,13]. Throughout their lifespan, cells are continuously exposed to endogenous stresses, unlike acute exogenous stresses.

To counteract the effects of genotoxic stresses, the DNA damage response (DDR) coordinates a network of metabolic pathways ensuring the faithful transmission of genetic material [5,6,14,15]. Schematically, activation of the DDR leads to the arrest of DNA synthesis and progression through the cell cycle, which gives time and full access to cofactors (e.g., ATP, nucleotides) to the DNA repair machinery; this allows the resumption of genomic replication on restored matrices. When DNA damage is too critical and the DNA repair systems are overwhelmed, senescence, apoptosis [14,16] or the innate immune system [17,18,19] are activated, avoiding the proliferation of cells bearing damaged genomes and genetic instability. Remarkably, the DDR has been shown to be activated in the pre- or early stages of senescence and tumorigenesis [20,21,22]. A defect in the DDR leads to increased sensitivity to genotoxic agents and genetic instability and is frequently associated with a predisposition to cancer and premature aging [20,21,22,23,24].

However, in the absence of exogenous stress, the progression of cells through the cell cycle is not stopped, and cells can replicate their genome despite chronic exposure to endogenous stresses. This suggests that the DDR is not (or is not fully) activated and that the stress intensity needs to pass a threshold for full activation of the DDR. This raises the question of whether cells actually respond to low-intensity stress or whether they have developed specific responses as alternatives to the canonical DDR (cDDR) mentioned above.

Recently, a noncanonical DDR specific to low stress in primary human cells, the low-level stress-DDR (LoL-DDR), which is different from the cDDR briefly mentioned above, was identified and characterized [25]. Moreover, this noncanonical response appears to be dysregulated in immortalized/transformed cell lines. Here, we describe this response in primary human cells and compare it with that in immortalized/transformed cell lines. We discuss the consequences for genome stability and cancer initiation and progression.

## 2. A Threshold of Stress Intensity for Full Activation of the cDDR in Primary Human Cells 

**Figure 1:**
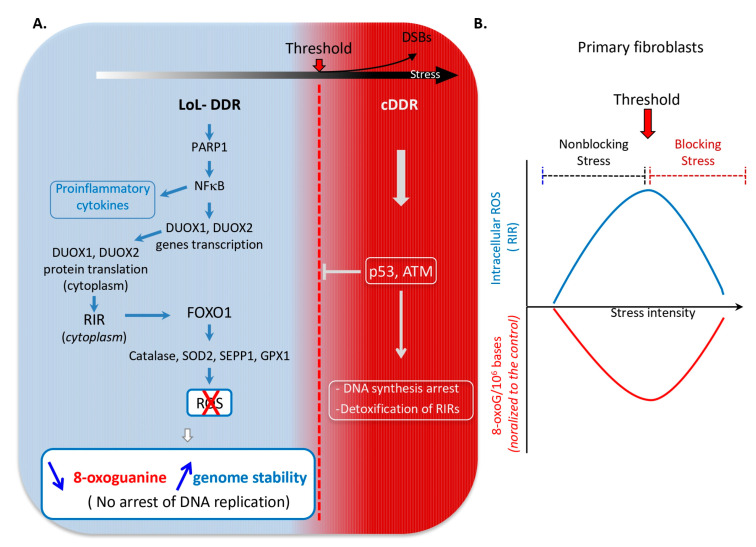
(**A**). The biphasic response model to DNA damage [25]. Primary cells adapt their response to the intensity of replication stress in two distinct phases: the low-level/endogenous stress response and the high-level stress response. Below a threshold of stress (red arrow), cells engage in a low-level response (LoL-DDR), which does not arrest DNA synthesis and cell cycle progression. The LoL-DDR regulates the production of extranuclear ROS (RIR) under cellular control via the following cascades: PARP1 activates NF-κB, which induces the expression of the *DUOX1* and *DUOX2* genes. Furthermore, NF-κB induces the expression of proinflammatory cytokines. Then DUOX1 and DUOX2 proteins are translated in the cytoplasm and produce ROS (RIR) in the cytoplasm. RIR produced by DUOX1 and DUOX 2 induce the detoxification program *FOXO1*, which protects against the accumulation of premutagenic lesions, such as 8-oxoguanine, in an adaptive-type detoxification response. Above this threshold, cells accumulate DSBs and activate the cDDR, which detoxifies RIR. (**B**). Effects of replication stress on RIR production (top blue line) and the accumulation of 8-oxoguanine (bottom red line) [25]. Below the stress threshold (red arrow), the cells are not blocked, and RIR accumulate; concomitantly, 8-oxoguanine levels in the genome decrease. Beyond the stress threshold (red arrow), RIR decrease, and 8-oxoguanine accumulation returns to basal levels.

Primary human skin fibroblasts and mammary epithelial cells adapt their responses according to the intensity of replication stress, delineating two distinct types of DDR (Figure 1) [25]. Primary cells were exposed to increasing concentrations of drugs that induce replication stress, such as hydroxyurea (HU, an inhibitor of ribonucleotide reductase that leads to an imbalance in the nucleotide pool), aphidicolin (APH, an inhibitor of replicative polymerases), or camptothecin (CPT, an inhibitor of topoisomerase I) [25] and ref in. The cDDR was fully activated only in response to the highest doses of these drugs, as monitored by the cell cycle distribution, DNA replication arrest, and p53 phosphorylation [25]. These findings suggest that a certain threshold of intensity of replication stress is required to fully activate the cDDR, which then leads to the arrest of DNA synthesis and the cell cycle in human primary fibroblasts. Remarkably, DNA double-strand breaks (DSBs) can be detected at the doses that induce the cDDR, as monitored by the phosphorylation of the histone variant γH2AX associated with p53 phosphorylation [25]. Below the stress threshold, cells continue to synthesize their genome and progress into the cell cycle. However, a noncanonical response different from the cDDR occurs, the LoL-DDR, which involves the cell-controlled production of reactive oxygen species (ROS) as secondary messengers and is specific to low levels of stress and to primary human cells (see below).

## 3. Cellular Regulation of the Low-Level Stress-DDR (LoL-DDR) in Primary Cells 

At the lowest stress intensity, below the threshold of activation of the cDDR, primary cells trigger a response that does not lead to replication arrest (nonblocking stress). This response is therefore specific to low-level intensity stress (LoL-DDR) in primary human cells. Paradoxically, the LoL-DDR produces cell-controlled ROS, which in turn protects genome stability in an adaptive way [25].

Upon nonblocking replication stress in human primary cells, the LoL-DDR proceeds via the following steps (Figure 1A) [25]: 1. PARP1, a DNA integrity sensor, activates the transcription factor NF-κB in a way that does not require PARP enzymatic activity. This implies that PARP1 can signal low levels of DNA damage; however, it is possible that other functions/roles of PARP1 may be involved. 2. NF-κB activates the transcription of the NADPH oxidases *DUOX1* and *DUOX2* genes [25]; then DUOX1 and DUOX2 proteins are translated into the cytoplasm and produce ROS. Note that physiologically generated ROS are involved during various fundamental cell metabolic processes [26]. In parallel, NF-κB induces the expression of proinflammatory cytokines [25]. 3. DUOX1 and DUOX2 synthesize cytoplasmic RIR (replication stress-induced ROS) [25]. Notably, the production of RIR is independent of p53 and ATM, indicating that this response fundamentally differs from that of the cDDR. 4. RIR activate the FOXO1-detoxifying program, which induces the expression of ROS-detoxifying genes, including *catalase*, *SOD2* (superoxide dismutase), *SEPP1* (selenoprotein P), and *GPX1* (glutathione peroxidase 1) [25]. 5. Induction of this ROS-detoxification pathway reduces the accumulation of premutagenic oxidized bases, such as 8-oxoguanine in DNA, thus protecting genome integrity from the genetic instability induced by oxidized bases [25]. Cytoplasmic ROS might affect the nucleotide pools, either through the oxidation of nucleotides or of the ribonucleotide reductase (RNR) that has been shown to be sensitive to oxidation [7]. However, since DUOX-induced ROS induces the FOXO1-detoxifying program, the activation of DUOX might in contrast prevent the oxidation of the nucleotide pools and of RNR, thus protecting genome stability.

Increasing the stress intensity above the threshold triggers cDDR activation, which leads to p53 phosphorylation and DNA replication arrest and detoxifies RIR via p53 and ATM (Figure 1A) [25]. As a result, the dose-response curve of RIR production is peak-shaped in primary cells (Figure 1B). This peak of RIR production precisely reveals the threshold at which the cDDR is activated. Remarkably, the curves for 8-oxoguanine accumulation as a function of stress intensity show a mirror profile compared with those for RIR production: a decrease in 8-oxoguanine accumulation at doses corresponding to the RIR peak, and 8-oxoguanine levels return to basal levels at higher doses, i.e., when RIR are abrogated (Figure 1B) [25]. Similar dose-response curves were obtained after different treatments, such as HU, APH, and CPT. Consistently, maintaining cell confluence, i.e., when DNA replication is abrogated, abolishes RIR production upon HU exposure, indicating that the production of such ROS actually results from DNA replication difficulties. Notably, the same type of peak-shaped curve was also observed in primary mammary epithelial cells [25]. Therefore, the cell response (LoL-DDR) appears to be a cell-controlled autonomous response, different from the cDDR, but is generalizable to nonblocking replication stress rather than to a specific drug and is found in primary cells from different tissues.

Note that phosphorylation of the H2AX variant (γH2AX), which mainly marks DSBs, is observed at the highest doses concomitant with cDDR activation. It is tempting to speculate that the detection and signaling of DSBs might also play a role in the induction of the transition from the LoL-DDR to the cDDR.

Finally, the LoL-DDR appears to be dysregulated in immortalized/transformed cell lines (see below).

Low doses of replication stress inducers (HU or APH) have been shown to slow replication without full arrest in immortalized/transformed cells [27,28]. Moreover, in B lymphocytes immortalized cells, and embryonic fibroblasts, stepwise activation of the signaling pathway has been described [27]. Note that replication and cell cycle arrest can be indirect effects of replication perturbation through the induction of the cDDR, which is frequently abnormal in transformed cells. However, one can object that a cell line might have dysfunctional cell cycle regulation and/or have a limited ability to reprime arrested replication forks [29]. Notably, in embryos, a “relaxed” control of genome integrity has been described, and the author concluded that this is reminiscent of observations in cancer cells; therefore, embryonic cells should behave differently from primary fibroblasts and epithelial cells [30,31]. Nevertheless, although immortalized and embryonic cells respond differently than primary cells to genotoxic stresses, even in these settings, the data also support the notion of a stress threshold for full activation of the cDDR [25,27].

## 4. Potential Role of the LoL-DDR in Competition Repair vs. Replication of DNA 

We propose that the LoL-DDR pathway helps cells cope with low-level replication stress. The cDDR, which requires a certain threshold of stress intensity before activation, arrests the cell cycle and DNA replication progression; therefore, cofactors such as ATP and nucleotides, which are needed for both DNA repair and replication, become fully available for repair. Below the stress threshold, since replication is not arrested but cells are being subjected to endogenous genotoxic stresses, competition for resources between DNA replication and DNA repair might occur. By reducing the level of endogenous damage (notably oxidative DNA damage), the LoL-DDR abolishes or attenuates this potential DNA repair/replication conflict, allowing for simultaneous management of both systems.

Increasing the level of stress intensity, and thus the level of DNA damage, could strongly unbalance the DNA repair/replication equilibrium, overwhelming the impact of DNA damage reduction mediated by the LoL-DDR. Note that a response that would further increase RIR levels would then become hazardous because of the potential detrimental effects of ROS, which can affect almost all types of biological molecules. Then, cDDR activation leads to cell cycle progression arrest and, concomitantly, RIR detoxification via ATM and p53.

## 5. LoL-DDR-Associated RIR Act in an Adaptive Way

Pre-treatment of cells with HU at doses that generate RIR also protects against ROS caused by exogenous exposure to 100 µM hydrogen peroxide (H_2_O_2_) [25]. This finding demonstrates that by triggering the FOXO1-detoxifying program through RIR induction, the LoL-DDR also detoxifies ROS from exogenous origins via an adaptive mechanism [25].

## 6. Activation of the FOXO1 Pathway in Leukemia Patients Treated with HU

Most patients suffering from chronic myelomonocytic leukemia (CMML), the most common myelodysplastic syndrome/myeloproliferative neoplasm, receive symptom-targeted treatments, such as HU, during the more proliferative stages of the disease [32]. HU treatment aims to reduce the number of circulating myeloid cells.

Notably, the concentrations of HU measured in patient serum are on the same order of magnitude as those used to induce RIR in primary fibroblasts in culture. Gene expression was analyzed in proliferative CD3-positive T lymphocytes and compared with that of nonproliferative CD14-positive monocytes in peripheral blood samples collected before and after the start of treatment. Consistent with the data obtained from cultured primary fibroblasts, FOXO1 target genes (*SEPP1*, *SOD2*, *GPX*, and *catalase*) were upregulated in CD3-positive T lymphocytes but not in CD14-positive monocytes [25]. These data confirm the activation of the FOXO1 ROS-detoxifying pathway by exposure of proliferating cells to HU in vivo and in a pathophysiological context.

## 7. LoL-DDR and Proinflammatory Cytokines 

In parallel with RIR production, the activation of NF-κB by nonblocking replicative stress induces the expression of genes encoding proinflammatory cytokines. When sustained, this can lead to inflammation and associated deleterious effects. However, these cytokines can also activate innate immunity, constituting an additional level of protection against genetic instability. Indeed, by eliminating cells with damaged DNA, innate immunity helps maintain global genomic stability.

Replication stress has been shown to induce proinflammatory cytokine production through the generation of cytosolic DNA, which activates the cGAS–STING pathway [17,18,19]. The existence of the LoL-DDR reveals that the production of inflammatory cytokines could be directly activated by the PARP1/NF-κB axis without necessarily requiring the generation of cytosolic DNA.

## 8. LoL-DDR Is Dysregulated in Immortalized/Transformed Cells at an Early Step of Cancer Progression

**Figure 2:**
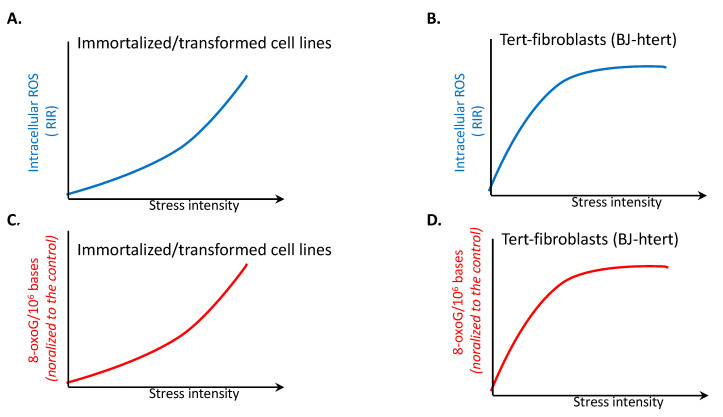
Accumulation of RIR and 8-oxoG in immortalized/transformed cell lines. (**A**)**.** Accumulation of RIR in immortalized/transformed cell lines. (**B**)**.** RIR accumulation in telomerase-immortalized human fibroblasts (BJ-hTERT). (**C**). 8-oxoG accumulation in immortalized/transformed cell lines. (**D**). 8-oxoG accumulation in telomerase-immortalized human fibroblasts (BJ-hTERT).

Transformed/immortalized cell lines respond differently than primary cells do when exposed to increasing doses of replication stress inducers [33]. ROS are indeed produced, but in contrast with primary cells, the dose-response curves are not peak shaped but continuously increase with increasing dose (compare Figure 2A with Figure 1B), suggesting that they are not detoxified at higher doses in transformed cells. These types of dose-response curves have been generated for different human cell lines, including SV40-transformed human fibroblasts, human colon cancer (RKO) cells, human osteosarcoma (U2OS) cells, and hamster cancer (V79) cells. Interestingly, cells immortalized with telomerase (hTERT) exhibit intermediate behavior, depending on the tissue of origin. Indeed, telomerase-immortalized retinal pigment epithelium (RPE) cells presented a dose-response curve with a continuous increase in RIR production similar to that of the cell lines listed above (Figure 2A), whereas in human telomerase-fibroblasts (BJ-hTERT), RIR levels plateaued at the highest dose (Figure 2B), which might correspond to an intermediate response between primary and transformed cells [33]. Nevertheless, these findings show that the LoL-DDR is altered in immortalized cells, with consequences that differ in cells of different tissue types.

Since the highest doses induce cDDR, this continuous increase in RIR could result from cDDR deficiency, as frequently observed in transformed cells; however, RIR production in transformed cell lines is independent of p53 status [33], and the cDDR is rather efficient in Tert-immortalized cells. Alternatively (or additionally), these progressive dose-response curves of RIR production at high replication stress doses could result from a high production level of ROS, which would result from LoL-DDR defects and thus the accumulation of ROS, eventually overwhelming the ROS-detoxifying systems.

Moreover, in transformed cells, RIR are resistant to diphenylene iodonium chloride (DPI), an inhibitor of all NADPH oxidases [34], in contrast with primary cells [25]. These findings indicate that NADPH oxidases, and thus neither DUOX1 nor DUOX2, are responsible for the production of RIR in transformed/immortalized cells.

Importantly, in contrast with primary cells, which show a decrease in 8-oxoG accumulation in the genome, transformed/immortalized cells exhibit a continuous increase in 8-oxoG accumulation with increasing dose (compare Figure 2C with Figure 1B), and the dose-response curves exhibit similar shapes to those of the RIR dose-response curves (compare Figure 2A and Figure 2B); notably, the accumulation of 8-oxoG reaches a plateau level at higher doses (Figure 2D), similar to RIR, in human tert-immortalized fibroblasts (BJ-hTERT) (compare Figure 2B and Figure 2D) [33].

Importantly, RIR have been shown to be extranuclear in primary cells [25]. This prevents unwanted attack on the genome, thus protecting genome stability. In contrast, transformed cell lines accumulate oxidative damage (8-oxoG) in the genome. This suggests that ROS are present in the nucleus or that nucleotides are oxidized in the cytoplasm before their transport into the nucleus and their incorporation into the genome in immortalized/transformed cell lines.

Table 1 summarizes the differences between primary and transformed/immortalized cells. Collectively, these data consistently indicate that the LoL-DDR is dysregulated in transformed/immortalized cells.

## 9. Replication Stress Generates a Form of Mitochondrial Stress That Produces Mitochondrial ROS

Since, in contrast with primary cells, RIR are not produced by NADPH oxidases in transformed cell lines, there must be other sources of ROS. Indeed, using MITOSOX (MitoSOX™ Red, Life Technologies, Waltham, MA, USA, cat#M36008), a probe specific for mitochondrial ROS, it has been shown that replication stress generates mitochondrial ROS, with continuously progressive dose-response curves [33]. Consistently, the mitochondrial membrane potential is altered as a function of the degree of replication stress [33]. Therefore, RIR in immortalized/transformed cell lines might correspond to mitochondrial ROS.

However, alterations in the mitochondrial membrane potential and the production of mitochondrial ROS, which progressively increase, are also detected in primary cells [33]. These findings suggest that at its highest doses, the cDDR does not detoxify mitochondrial ROS. However, when global intracellular ROS are monitored via CM-H2DCFDA or DHR 123 probes, primary cells exhibit peak-shaped curves, indicating that global ROS are detoxified at higher doses [25]. Since, owing to the DUOX1- and DUOX2-produced RIR, the LoL-DDR induces the FOXO1-detoxifying program, which is capable of detoxifying even exogenous ROS [25], one can propose that it is also able to control and limit the accumulation of mitochondrial ROS in primary cells. This capability is deactivated in immortalized/transformed cell lines, which is consistent with the dysregulation of the LoL-DDR in such cells.

## 10. LoL-DDR Is Dysregulated at an Early Step of Cancer Progression in an In Vitro Model

The fact that the LoL-DDR appears to be dysregulated in telomerase-transformed cells suggests that it might be altered in an early step of cancer progression. To address this hypothesis, an in vitro cell culture model of breast cancer progression was used [33]. This model was established with primary epithelial cells (HUMECs), telomerase-immortalized HUMECs (HME-hTERT cells), HME-hTERT cells expressing the SV40 large T antigen (HMLE-SV40), and HMLE-SV40 cells expressing the V-RAS oncogene [35,36]. RIR peaked in primary epithelial breast cells at the same dose as in primary fibroblasts. However, in the HMR-hTERT cells (similar to RPE-hTERT cells), the dose-response curves lost their peak shape with increasing dose, similar to what was observed in the other transformed cell lines [33]. Moreover, the RIR produced in this cell system were resistant to DPI, indicating that they were not produced by an NADPH oxidase. These findings suggest potential dysregulation of the LoL-DDR in the early stage of breast cancer progression.

However, this model is an extrapolation of the actual situation in vivo; thus, experiments in other systems and in tumors need to be performed to validate this important hypothesis.

## 11. LoL-DDR and Cancer

The LoL-DDR might represent an important pathway for cancer prevention. First, while most NADPH oxidases are upregulated in cancer, in contrast, only DUOX1 and DUOX2 (which play a central role in the LoL-DDR) are lost in many different tumor types [37], suggesting that the LoL-DDR has also been lost in these tumors. These findings demonstrate the protective effects of ROS (RIR) controlled by DUOX1 and DUOX2. Remarkably, antioxidants, which are also able to detoxify RIR [25], have been suggested to protect against carcinogenesis. However, in contrast, these agents promote lung carcinoma and melanoma metastasis [38,39]. These findings highlight the potential beneficial role of controlled ROS in human cell homeostasis.

Consistent with a tumor inhibiting role of the LoL-DDR, defects in each of the players of the LoL-DDR, such as NF-κB, PARP1, FOXO1, DUOX1, and DUOX2, lead to common phenotypes, including alterations in cellular homeostasis/metabolism, aging, and cancer predisposition. These data are consistent with the fact that, in an in vitro breast cancer model, the LoL-DDR seems altered at an early step of cancer progression. Although additional experiments are required, the LoL-DDR might represent an enticing candidate to design diagnostic tests identifying tumors in early stages, thus allowing optimization of the efficiency of cancer therapy.

## 12. Concluding Remarks and Perspectives

Collectively, these data reveal a fine-tuned cell autonomous response adapted to the intensity of replication stress in primary human cells. At stress levels that only weakly affect the cell cycle distribution and DNA synthesis or do not affect it at all, a specific noncanonical response that is dedicated to low-level replication stress, the LoL-DDR, is triggered [25]. The LoL-DDR should thus be involved in the response to endogenous stresses. Since cells are chronically exposed to low-intensity endogenous stresses throughout their life, in contrast to acute exposure to severe stress, the LoL-DDR is expected to play a key role in maintaining genome integrity in primary human cells. Consequently, the precise and appropriate regulation of these processes is essential to protect the genome against these pernicious and unavoidable daily threats.

**Figure 3:**
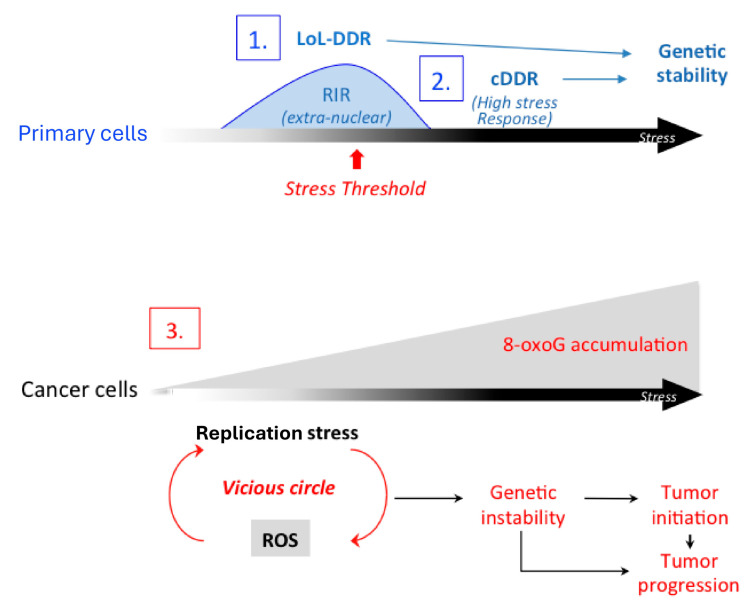
Networks of ROS/replication stress in primary versus cancer cells.

The model presented in Figure 3 assembles and unifies the different data: 1- In primary noncancerous cells, nonblocking low-level replication stress induces the LoL-DDR, which protects genome stability through the induction of ROS. Whether the LoL-DDR also responds to other kinds of genotoxic stresses (ionizing radiation, UV, etc.) should be examined. Since ROS represent a potential threat to all biological entities and can particularly jeopardize genome integrity, they should be tightly controlled; indeed, ROS production is under the precise control of the cellular enzymes DUOX1 and DUOX2, which are themselves regulated by cellular enzymes (PARP1 and NF-κB); importantly, these ROS are cytoplasmic and excluded from the nucleus, thus preventing any attack on the genome. These ROS then induce the FOXO1-detoxifying program, protecting against endogenous and exogenous ROS in an adaptive way. This cell autonomous pathway thus protects genome integrity from oxidative damage that can lead to genetic instability. 2- Increased stress intensity induces the cDDR, and ATM and p53 turn off the LoL-DDR. Therefore, the LoL-DDR could be considered a pre-cDDR, but the two pathways are different. The cDDR also maintains genetic stability but in a different way than the LoL-DDR does. Deficiency in the cDDR confers cancer predisposition [5,6,14,15,40]; similarly, all the factors that play pivotal roles in controlling the LoL-DDR (NF-κB, PARP1, FOXO1, DUOX1, and DUOX2) are lost in multiple types of tumors [37,41,42,43,44,45,46], suggesting that, like cDDR factors, although they are different, they might also protect against cancer. 3- In immortalized cells, the LoL-DDR is dysregulated, presumably in an early stage of cancer progression; replication stress induces ROS production even at high levels of stress, leading to the accumulation of premutagenic lesions in the genome. Conversely, ROS cause replication stress [7,9], triggering a vicious cycle of ROS/replication stress, which maintains and amplifies genetic instability and thus plays an important role in cancer etiology in both the initiation and progression stages (Figure 3).

Several questions remain to be explored to obtain a more detailed molecular landscape of the LoL-DDR. For example, the ATR/CHK1 axis plays an essential role in controlling the cDDR following replication stress. Since the described LoL-DDR also acts after replication stress [25,33], the potential role of ATR/CHK1 remains to be addressed. One hypothesis is that ATR/CHK1 acts both in the LoL-DDR and in the cDDR. Proving this hypothesis faces experimental difficulty. Indeed, ATR inhibition aggravates replication stress intensity [47], and high replication abolishes RIR production [25]; therefore, when ATR/CHK1 is inhibited, if LoL-DR decreases, it is difficult to distinguish whether this phenomenon would actually result from direct inhibition of the LoL-DDR pathway or from an increase in replication stress intensity. Another important question that remains to be elucidated is the mechanism underlying the shift from the LoL-DDR to the cDDR. DSBs might play a role, but if so, the mechanism is unclear. More generally, the potential impact of actors of the cDDR other than p53 and ATM on LoL-DDR should be evaluated.

It has been shown that primary human fibroblasts exposed to 10 mGy, i.e., a low dose of ionizing radiation, fail to repair DSBs, suggesting that a threshold of IR should be reached for efficient repair. Remarkably, pre-treatment of cells with 10 µM H_2_O_2_ restored repair efficiency [48]. Determining if the path (PARP1/NF-κB/DUOX1,2/Foxo1) is involved after IR and replication stress, i.e., LoL-DDR, is essential. Notably, is the induction of the ROS-detoxifying program also involved following low doses of radiation? This could be important for radiation therapy during which bystander cells can be affected by lesions corresponding to low doses, leading to deleterious effects such as the initiation of secondary cancers. If the induction of the LoL-DDR, as an adaptive response, can actually protect against such effects, this might constitute an enticing strategy to optimize radiation therapy.

Since the majority of cell models used in laboratories are cell lines (for convenience reasons) in which the LoL-DDR is altered, even in telomerase-immortalized cell lines [33], determining how the LoL-DDR is dysregulated in cell lines and in tumors at the molecular level is essential. For example, are the main actors of the LoL-DDR (PARP1, NF-κB, NADPH oxidases, and FOXO1) altered in immortalized/transformed cell lines and in tumors?

In primary cells, RIR are produced in the cytoplasm and excluded from the nucleus, preventing premutagenic genome injuries such as 8-oxoG [25]. In contrast, 8-oxoG accumulates in immortalized/transformed cell lines upon replication stress [33]. These findings suggest that, in contrast with primary cells, ROS are present in the nucleus. Considering the very short half-life of ROS and their high reactivity, one question is how ROS accumulate in the nucleus, leading to genome assault in immortalized/transformed cell lines, and whether these processes are also at work in tumors. Alternatively, nucleotides might be oxidized in the cytoplasm and then transported into the nucleus and incorporated into the genome. This hypothesis implies that primary cells would be protected from this process through the induction of the FOXO1 pathway, which would limit the accumulation of oxidized nucleotides, but not in immortalized/transformed cell lines.

Using an in vitro cell model, the data suggest that the LoL-DDR might be altered at an early step of cancer progression. This question should be addressed in experimental systems closer to the pathology. An important issue would be the development of markers of LoL-DDR dysfunction into early diagnostic tests for carcinogenesis.

More generally, deciphering the impact of the LoL-DDR on carcinogenesis and aging is an exciting challenge with excellent future prospects.

## Figures and Tables

**Table 1:** Differences between primary cells and immortalized/transformed cell lines in response to replication stress.

	Primary Cells	Immortalized/Transformed Cell Lines
Replication stress dose-response curve	Peak shape	Continuous progressive
NADPH-oxidase requirement	DUOX1 DUOX2	−
8-oxoG accumulation	Protected	+
Nuclear ROS	Excluded	Presumably present
